# Pathological Study on Epithelial-Mesenchymal Transition in Silicotic Lung Lesions in Rat

**DOI:** 10.3390/vetsci6030070

**Published:** 2019-08-30

**Authors:** Mao Komai, Karin Mihira, Akinori Shimada, Ikumi Miyamoto, Kikumi Ogihara, Yuko Naya, Takehito Morita, Kenichiro Inoue, Hirohisa Takano

**Affiliations:** 1 Laboratory of Pathology, School of Life and Environmental Science, Azabu University, 1-17-71 Fuchinobe, Sagamihara-shi, Kanagawa 252-5201, Japan; 2 Department of Veterinary Pathology, Tottori University, 4-101 Koyama Minami, Tottori-shi, Tottori 680-8553, Japan; 3 School of Nursing, University of Shizuoka, Shizuoka-shi, Shizuoka 422-8526, Japan; 4 Department of Environmental Engineering, Kyoto University Graduate School of Engineering, Kyoto-shi, Kyoto 615-8530, Japan

**Keywords:** epithelial-mesenchymal transition, fibrosis, rat, silicosis, TGF-β, type II epithelial cells

## Abstract

Silicosis, caused by the inhalation of crystalline silicon dioxide or silica, is one of the most severe occupational diseases. Persistent inflammation and progressive massive pulmonary fibrosis are the most common histological changes caused by silicosis. Association of epithelial-mesenchymal transition (EMT) of hyperplastic type II epithelial cells with the fibrotic events of pulmonary fibrosis has been suggested in in vitro silica-exposed cultured cell models, patients with idiopathic pulmonary fibrosis, and bleomycin-induced experimental models. Histological features of EMT, however, are not fully described in silicotic lungs in in vivo. The purpose of this study was to demonstrate EMT of hyperplastic type II epithelial cells in the developmental process of progressive massive pulmonary fibrosis in the lungs of rats exposed to silica. F344 female rats were intratracheally instilled with 20 mg of crystalline silica (Min-U-Sil-5), followed by sacrifice at 1, 3, 6, and 12 months after instillation. Fibrosis, characterized by the formation of silicotic nodules, progressive massive fibrosis, and diffuse interstitial fibrosis, was observed in the lungs of the treated rats; the effects of fibrosis intensified in a time-dependent manner. Hyperplasia of the type II epithelial cells, observed in the massive fibrotic lesions, dominated in the lungs of rats at 6 and 12 months after the treatment. Immunohistochemistry of the serial sections of the lung tissues demonstrated positive labeling for cytokeratin, vimentin, and α-smooth muscle actin in spindle cells close to the foci of hyperplasia of type II epithelial cells. Spindle cells, which exhibited features of both epithelial cells and fibroblasts, were also demonstrated with bundles of collagen fibers in the fibrotic lesions, using electron microscopy. Increased expression of TGF-β was shown by Western blotting and immunohistochemistry in the lungs of the treated rats. These findings suggested that enhanced TGF-β expression and EMT of hyperplastic type II epithelial cells are involved in the development process of progressive massive pulmonary fibrosis during silicosis.

## 1. Introduction

Silicosis, caused by the inhalation of crystalline silicon dioxide or silica, is one of the most severe occupational diseases [1,2]. Persistent inflammation and pulmonary fibrosis are the most common histological changes during silicosis [3,4,5]. Accumulating evidence indicates that silica particles activate macrophages and epithelial cells, causing them to release copious amounts of oxidants and cytokines, which, in turn, leads to fibroblast proliferation, epithelial-mesenchymal transition (EMT), deposition of extracellular matrix, and ultimately, fibrosis (silicosis) [1,6,7]. Although the pathogenic factors of silicosis are already established, the complex biological and molecular mechanisms underlying silicosis have not yet been fully elucidated [1,7].

EMT is a process in which epithelial cells gradually acquire a mesenchymal (fibroblast-like) cell phenotype [8]. EMT has been shown to be essential for embryonic development, gastrulation, and the development of the neural crest, heart, and other organs [9,10]. EMT is also implicated in tissue repair, organ fibrosis, and cancer progression [11,12]. 

TGF-β exerts multiple effects that may exacerbate fibrosis. There is a consistent increase in TGF-β production in epithelial cells and macrophages in lung tissue from patients with idiopathic pulmonary fibrosis (IPF) [13]. Transient overexpression of active TGF-β through the transfection of porcine TGF-β cDNA into the rat lung results in prolonged and severe interstitial and pleural fibrosis [14]. Induction of EMT by TGF-β on a primary culture of human bronchial [15,16] and human [17] and rat [18] alveolar epithelial cells were demonstrated. 

Dual-immunohistochemistry demonstrated co-expression of epithelial and mesenchymal proteins in lung tissues in patients with IPF and bleomycin-induced experimental models [18,19]. Accordingly, EMT is likely involved in the pathogenesis of pulmonary fibrosis, and TGF-β is also considered to play an important role in this process [18]. Association of EMT of hyperplastic type II epithelial cells with the fibrotic events in pulmonary fibrosis has been suggested in in vitro silica-exposed cultured cell models, patients with idiopathic pulmonary fibrosis, and bleomycin-induced experimental models [18]. Histological features of EMT, however, are not fully described in in vivo, and there is a lack of evidence for the association of EMT with the pathophysiology of silicotic pulmonary fibrosis.

The purpose of this study was to demonstrate the role of EMT of hyperplastic type II epithelial cells in the development process of progressive massive pulmonary fibrosis in the lungs of rats exposed to silica.

## 2. Materials and Methods 

### 2.1. Animals

A total of 56 female, 6-week-old F344 rats were obtained from CLEA JAPAN (Tokyo, Japan). The animals were fed a diet, CE-2, purchased from CLEA JAPAN, and water was given ad libitum. The rat cages were placed in a conventional room, where the temperature was maintained at approximately 25 °C and the humidity was maintained at 55% to 70%. All experiments were performed according to the guidelines of The Laboratory Animal Care Committee of Azabu University (#170412-2: http://kitei.azabu-u.ac.jp/reiki/Re05_Hon_Main_Frame.exe?UTDIR=D:\EFServ2\ss000001DD\reiki&TID=2&SYSID=1331). Changes in body weight were recorded every week to assess the general health of the rats. 

### 2.2. Preparation of Silica Particle Suspensions

Crystalline silica particles (Min-U-Sil-5, U.S. Silica Co., Frederick, MD, USA), silicon dioxide (SiO_2_), with an average diameter of 1.6 μm were used in this study. The particles were hot-air-sterilized at 300 °C for 1 h to remove toxic substances (microbiological substances and chemicals, such as nitrogen oxide and sulfur oxide) adhering to the particles. The particles were suspended in 0.05 mL of sterilized 0.9% NaCl solution for instillation. Particles with a total mass of 20.0 mg were selected to determine the effects on lung toxicity in rats. The maximum deposition of particles in the lungs of a single rat was calculated using tidal volume and breathing rate [20]. The maximum weekly deposition of suspended particulate matter (0.1 mg/m^3^), as measured by the Japanese National Air Quality Standard, was approximately 0.03 mg. The instillation dose (20.0 mg) in the present study represents 666 times that amount [21]. Control rats were administered 0.05 mL of sterilized 0.9% NaCl solution.

### 2.3. Study Protocol

The rats were randomly divided into four control (*n* = 6 each) and four exposure (*n* = 8 each) groups. The rats were anesthetized using an i.p. injection of sodium pentobarbital (5 mg/100 g body weight) (Wako, Osaka, Japan). The suspensions were agitated just prior to intratracheal instillation, and 0.05 mL of the suspension was instilled in each rat using an intratracheal cannula. Each intratracheal instillation procedure took 3 s.

The rats in each of the four groups were euthanized by exsanguination under deep anesthesia induced by i.p. injection of sodium pentobarbital at 1, 3, 6, and 12 months after instillation, respectively. 

### 2.4. Pathological Examination

Three rats from each control group and three rats from each exposure group were used for gross and histological examinations, and immunohistochemistry. A complete necropsy examination was performed. After gross examination, the lungs were removed and weighed, and the ratio of the lung weight/body weight was calculated. The heart, liver, spleen and kidney were also taken for histology. The lung lobes were separated, and longitudinal sections from each lobe were prepared. Selected tissues from the heart, liver, spleen, kidney and the lung tissue sections were placed into embedding cassettes and fixed by immersion in 10% neutral-buffered formalin. The formalin-fixed tissues were routinely processed and embedded in paraffin for histopathological and immunohistochemical examination. Sections of the tissues (approximately 3 μm thick) were cut and stained with hematoxylin and eosin (H&E), Masson’s trichrome (MT) and Periodic acid Schiff (PAS) stain.

### 2.5. Immunohistochemistry

Paraffin-embedded sections of the lungs treated with saline or with 20.0 mg of silica particles were used for the immunohistochemical detection of cytokeratin K8/K18, vimentin, *α*-smooth muscle actin, TGF-β and Ki-67. A semiquantitative evaluation of the immunohistochemical reaction of TGF-β and Ki-67 was conducted by counting the number of stained cells in 10 microscopic fields at 400× magnification. This step was performed five times and the mean values were calculated and recorded. Serial sections were used to identify the location of the two sets of positive reactions in the cytoplasm of the hyperplastic epithelial cells (cytokeratin and vimentin) and spindle cells (cytokeratin and *α*-smooth muscle actin) in the fibrotic lesions. For antigen retrieval, the sections were placed in a citrate buffer solution (pH 5.4) and microwaved for 20 min. Endogenous peroxidase activity was quenched by treatment with 3% H_2_O_2_ for 30 min at room temperature (RT). Slides were then incubated with 10% normal goat serum for 1 h at RT in order to block non-specific antibody binding. Thereafter, the sections were incubated with primary antibodies overnight at 4 °C. Antibodies used were anti-cytokeratin K8/K18 (Progen, Heidelberg, Germany), at 1:200 dilution; anti-vimentin (Dako, Glostrup, Denmark), at 1:50 dilution; anti-*α*-smooth muscle actin (R&D Systems, Minneapolis, MN, USA), at 1:200 dilution; anti-TGF-β (Bioss, Boston, MA, USA), at 1:1000 dilution; and anti-Ki-67 (Proteintech, Rosemont, IL, USA) at 1:2000 dilution. The primary antibodies were replaced with phosphate-buffered saline (PBS) in negative controls. After incubation with primary antibodies, the sections were placed in a solution containing a peroxidase-labeled polymer conjugated to a secondary anti-rabbit antibody (EnVision + kit/HRP (DAB), (Dako)) for 30 min at RT. Positive regions were stained brown using the chromogen 3,3′-diaminobenzidine tetrahydrochloride (DAB, Wako). Then, the sections were counterstained with hematoxylin. 

### 2.6. Statistical Analysis

Statistical analysis of the ratio of the lung weight/body weight, semiquantitative immunohistochemistry and Western blot was performed using a Welch’s *t*-test and a Bonferroni’s multiple comparison test. For all comparisons, *p* values less than 1% (*p* < 0.01) and 5% (*p* < 0.05) were considered statistically significant.

### 2.7. Transmission Electron Microscopy

Among the rats of each group that were sacrificed 12 months after treatment, half of the longitudinal sections of each lung lobe were used for transmission electron microscopy. Cubes of 1–2 mm^3^ were prepared from each section. They were fixed in 2.5% glutaraldehyde for 3 h at 4 °C, rinsed in 0.1 M phosphate buffer (pH = 7.4), fixed for 1 h in 1% osmium tetroxide, dehydrated in alcohol, and embedded in epoxy resin. Semi-thin (1 μm) sections were stained using 1% toluidine blue. Ultra-thin sections stained with uranyl acetate and lead citrate were then examined under a JEOL JEM 2100 electron microscope (Tokyo, Japan).

### 2.8. Western Blot Analysis

Three rats from the control group sacrificed 6 months after saline treatment and three rats from each exposure group were used for Western blot analysis. The frozen lung tissues were lysed with a RIPA lysis buffer (50 mM Tris-Cl (pH 7.6), 150 mM NaCl, 1% NP-40, 0.1% SDS, 0.5% deoxycholic acid, 1 µg/mL leupeptin, 1 µg/mL aprotinin, and 0.5 mM phenylmethylsulfonyl fluoride) and were centrifuged at 12,000× *g* at 4 °C for 30 min to obtain the cellular proteins in the supernatant. Equal amounts of proteins from each sample were resolved by SDS-PAGE, transferred to NC membranes, blocked with 5% skimmed milk for 1 h at 25 °C, and probed at 4 °C overnight with anti-TGF-β primary antibody (Bioss) at 1:1000 dilution, and anti-α-smooth muscle actin primary antibody (R&D Systems) at 1:200 dilution. Blots were subsequently probed with horseradish peroxidase-conjugated anti-rabbit IgG (Dako, Glostrup, Denmark) at 1:1000–5000. Immunoreactive bands were visualized by enhanced chemiluminescence. The intensity of the bands was analyzed using ImageJ (http://imagej.nih.gov/ij/).

## 3. Results

**Figure 1:**
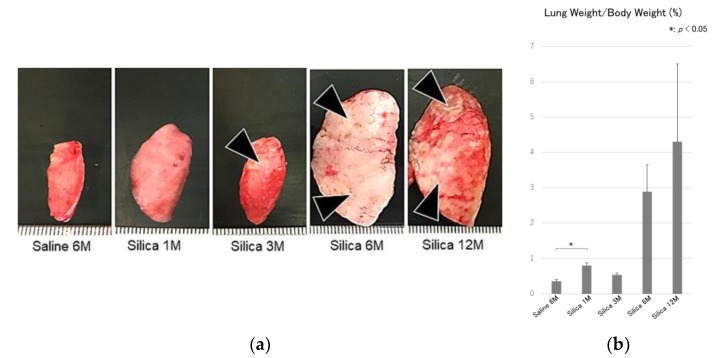
(**a**) Gross findings of the lung. Enlargement with an increase in the size of the organ, together with discoloration characterized by the formation of multifocal to confluent whitish, firm nodules (black arrow heads) are shown in the treated lungs. The change is prominent at 6 and 12 months after silica treatment. Saline 6M: 6 months after saline treatment (control), Silica 1M: 1 month after silica treatment, Silica 3M: 3 months after silica treatment, Silica 6M: 6 months after silica treatment, Silica 12M: 12 months after silica treatment. M: Month, and (**b**) ratio of the lung weight/body weight, showing increased ratio after silica treatment. M: month. All data are expressed as mean ± SD. Statistically significant differences (* *p* < 0.05) are indicated.

To examine the pathological features of EMT in silica-mediated pulmonary fibrosis, rats were treated with crystalline silica and sacrificed at 1, 3, 6, and 12 months after the treatment. In lung samples, enlargement with an increase in both the size and weight of the organ, together with discoloration, were observed in the treated lungs. Multifocal to confluent whitish, firm nodules were evident (Figure 1a,b). The change was prominent at 6 and 12 months after silica treatment.

**Figure 2:**
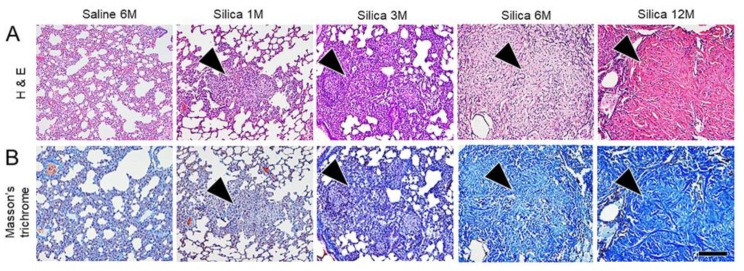
Microscopic findings of the serial lung tissue sections stained with hematoxylin and eosin stain (H & E) (**A**) and Masson’s trichrome (**B**). Time-dependent fibrosis characterized by progressive massive fibrosis with the formation of silicotic nodules (arrow heads) is observed in the lung of the treated rats. Bar = 150 μm. M: Month.

**Figure 3:**
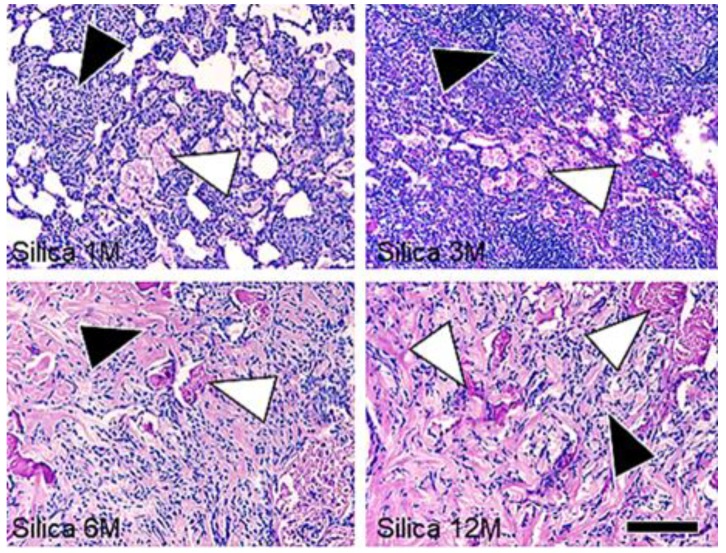
Microscopic findings of the fibrotic lesions of the lung tissue sections stained with Periodic acid Schiff stain. Proteinosis (white arrow heads) adjacent to the fibrotic lesions (black arrow heads) is observed in the lung of the treated rats. Bar = 150 μm. M: Month.

Time-dependent fibrosis, characterized by the formation of silicotic nodules consisting of granuloma, adjacent type II epithelial cell hyperplasia, fibroblast proliferation with bundles of interlacing collagen fibers, progressive massive fibrosis, and diffuse interstitial fibrosis, was observed in the lungs of the treated rats (Figure 2). Multifocal proteinosis was demonstrated adjacent to the fibrotic areas (Figure 3).

Animals sacrificed at 6 and 12 months showed moderately dilated hearts containing small foci of hypertrophy and degeneration and loss of cardiac myofibers with fibrosis, in addition to the severe pulmonary fibrosis.

**Figure 4:**
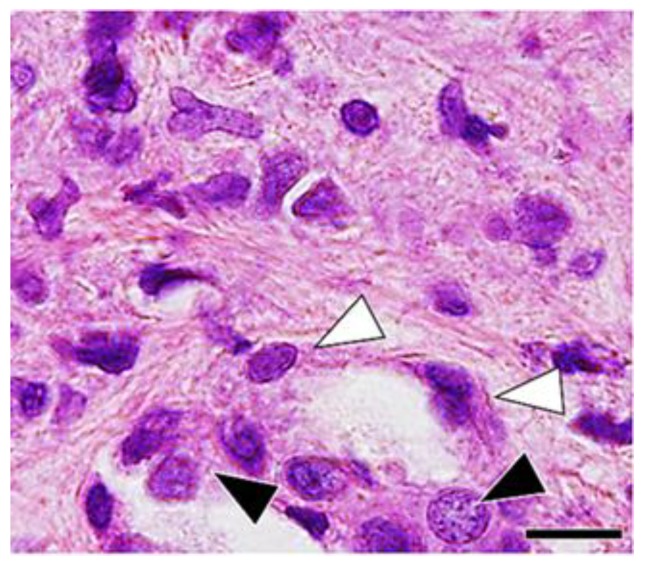
Microscopic findings of the fibrotic lesion of the lung tissue at 12 months after silica treatment, showing hyperplasia of type II epithelial cells (black arrow heads) with frequent appearance of spindle and elongated shape (white arrow heads). Hematoxylin and eosin. Bar = 20 μm.

**Figure 5:**
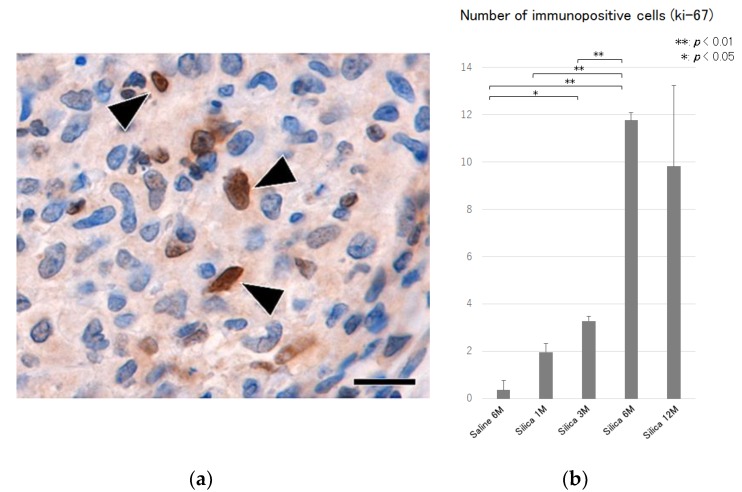
(**a**) Microscopic findings of the fibrotic lesion of the lung tissue at 6 months after silica treatment, showing positive labeling for Ki-67 in spindle cells (black arrow heads) in the fibrotic lesions. Ki-67 immunohistochemistry. Bar = 15 μm, and (**b**) semiquantitative analysis of the immunohistochemical reactions of Ki-67 in the lung tissues. An increase in the number of Ki-67 positive cells are shown in the treated lungs. All data are expressed as mean ± SD. Statistically significant differences (* *p* < 0.05, ** *p* < 0.01) are indicated.

**Figure 6:**
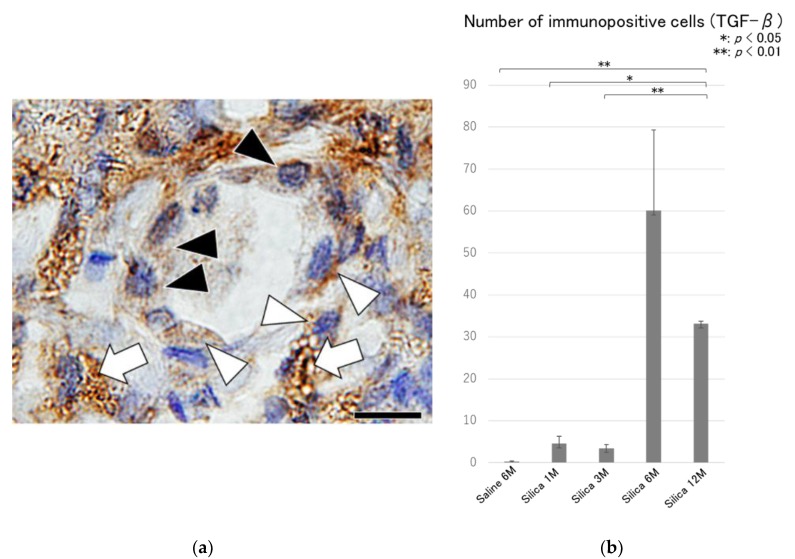
(**a**) Microscopic findings of the fibrotic lesion of the lung tissue at 6 months after silica treatment, showing TGF-β positive labelling in macrophages (white arrows), hyperplastic epithelial cells (black arrow heads) and spindle cells (white arrow heads). TGF-β immunohistochemistry. Bar = 20 μm, and (**b**) semiquantitative analysis of the immunohistochemical reactions of TGF-β in the lung tissues. Increase in the number of TGF-β positive cells is shown in the treated lungs. All data are expressed as mean ± SD. Statistically significant differences (* *p* < 0.05, ** *p* < 0.01) are indicated.

Hyperplasia of the type II epithelial cells with frequent appearance of spindle and elongated shapes were observed in the massive fibrotic lesions (Figure 4), and an increase in the number of Ki-67 immunoreactivity (Figure 5) were observed in the massive fibrotic lesions, and these effects were prominent in the lungs of rats at 6 and 12 months after the treatment. TGF-β immunohistochemistry demonstrated positive labeling in macrophages, epithelial cells, and spindle cells in the fibrotic lesions (Figure 6). 

**Figure 7:**
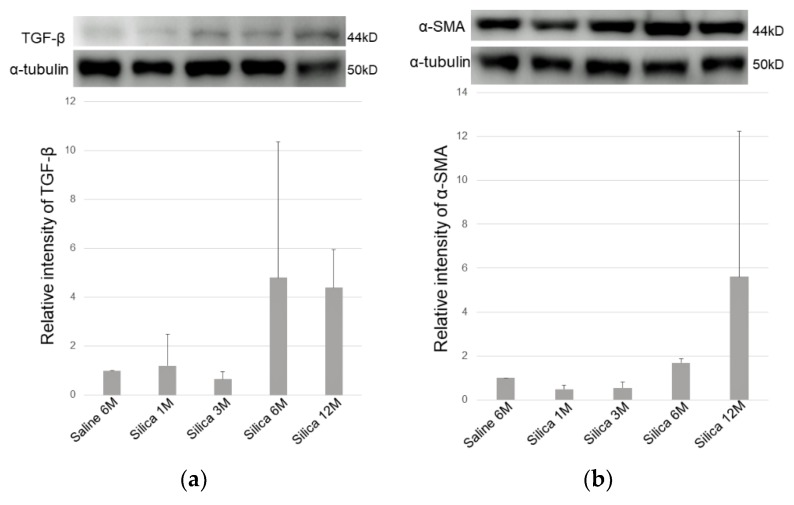
(**a**) Protein expression of TGF-β and (**b**) *α*-smooth muscle actin examined by Western blotting analysis. Increased TGF-β and *α*-smooth muscle actin positivity is shown in the silica treated lung tissues. M: month. All data are expressed as mean ± SD.

Western blotting revealed upregulation of TGF-β and *α*-smooth muscle actin proteins after silica exposure in a time-dependent manner (Figure 7a,b). 

**Figure 8:**
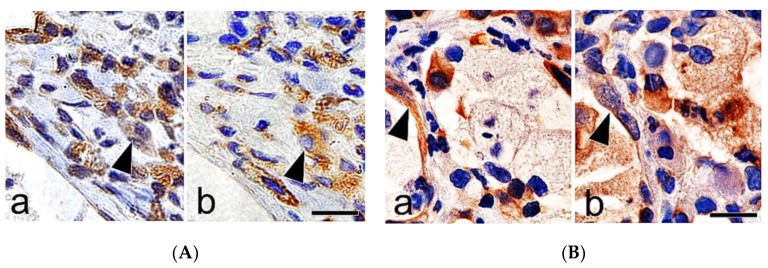
(**A**) Immunohistochemistry of the serial sections of the lung tissues at 12 months after silica treatment, showing cytokeratin (**a**: a black arrow head) and vimentin (**b**: a black arrow head) positive labelling in hyperplastic epithelial cells in the fibrotic lesions. Bar = 15 μm, and (**B**) immunohistochemistry of the serial sections of the lung tissues at 12 months after silica treatment, showing cytokeratin (**a**: a black arrow head) and *α*-smooth muscle actin (**b**: a black arrow head) positive labelling in spindle cells in the fibrotic lesions. Bar = 15 μm.

Immunohistochemistry of the serial sections of the lung tissues demonstrated positive labeling for cytokeratin, vimentin, and *α*-smooth muscle actin in spindle cells close to the foci of hyperplasia of type II epithelial cells (Figure 8A,B). 

**Figure 9:**
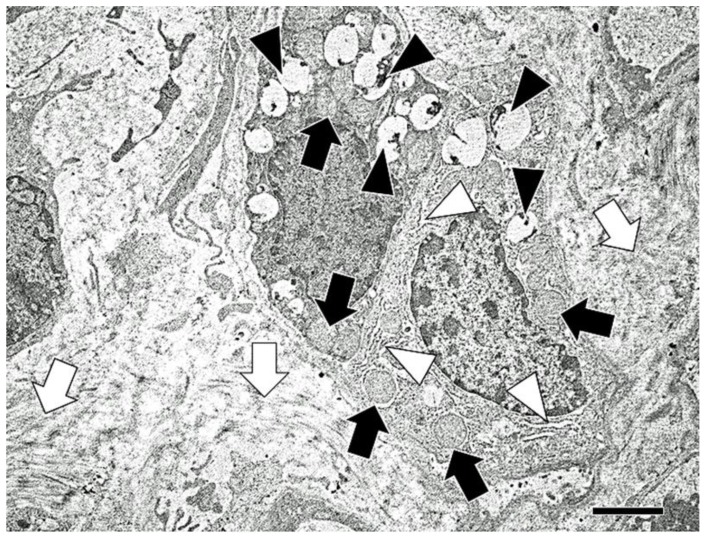
Electron microscopy of the lung tissues at 12 months after silica treatment. Elongated cells, which have features of alveolar type II epithelial cells (lamellar bodies) (white arrow heads) contain abundant rough endoplasmic reticulum (black arrow heads) and mitochondria (black arrows), are shown in the fibrotic lesions with increased collagen bundles (white arrows). Note no desmosome structures between the two cells. Bar = 5 μm.

Elongated cells, which have lamellar bodies (features of alveolar type II epithelial cells) and abundant rough endoplasmic reticulum and mitochondria (features of fibroblasts) were demonstrated in the fibrotic lesions, using electron microscopy (Figure 9).

## 4. Discussion

Silicosis, an occupational disease caused by inhaling silica, is characterized by progressive pulmonary fibrosis [1,3,4,5,6]. Pathological varieties of silicosis include simple (nodular) silicosis, progressive massive fibrosis, silicoproteinosis, diffuse interstitial fibrosis, and gross pathological examination of the affected lung shows discrete hard nodules [1,5]. These findings were consistent with our results in the present study conducted in a rat model. 

Consistent increase in TGF-β production in epithelial cells and macrophages was demonstrated in lung tissue obtained from patients with IPF [13]. In a bleomycin-induced pulmonary fibrosis model, TGF-β is expressed in alveolar macrophages during the acute phase of inflammatory cell infiltration, and in epithelial cells at later stages of pulmonary fibrosis [22]. TGF-β also mediates fibroblast proliferation and the differentiation of type II epithelial cells into fibroblasts, and plays an essential role in the development of pulmonary fibrosis [23]. Immunohistochemical localization of TGF-β was studied in the lungs of rats exposed to crystalline silica [24] and positive reactivity was demonstrated in hyperplastic type II cells adjacent to the fibrotic lesions. In this study, TGF-β positive labeling was also demonstrated in macrophages, epithelial cells, and spindle cells present in the fibrotic lesions, and time-dependent increase in TGF-β expression was associated with these changes. Thus, TGF-β may have multiple effects that may exacerbate fibrosis [24].

EMT is a process in which epithelial cells gradually acquire a mesenchymal (fibroblast-like) cell phenotype [8]. Association of EMT of hyperplastic type II epithelial cells with the fibrotic events in pulmonary fibrosis has been suggested in in vitro silica-exposed cultured cell models, patients with IPF, and bleomycin-induced experimental models [18]. Histological features of EMT, however, are not fully described in silicotic lungs in in vivo. Yamada et al. [25] reported that there was little direct evidence of EMT in the pulmonary fibrosis based on the results of dual-immunohistochemistry on lung tissues from bleomycin-induced pulmonary fibrosis in mice, and from patients with IPF. In this study, immunohistochemistry of the serial sections of the lung tissues demonstrated positive labeling for cytokeratin, vimentin, and α-smooth muscle actin in spindle cells close to the foci of hyperplasia of type II epithelial cells. Spindle cells, which exhibited features of both epithelial cells and fibroblasts, were also demonstrated with bundles of collagen fibers in the fibrotic lesions, using electron microscopy. In addition, increased expression of TGF-β and *α*-smooth muscle actin was confirmed by Western blotting and immunohistochemistry in the lungs of the treated rats. These findings suggested that enhanced TGF-β expression and EMT of hyperplastic type II epithelial cells are associated with the development process of progressive massive pulmonary fibrosis during silicosis [18]. Attenuation of TGF-β expression, and resultant downregulation of EMT, could be a potential therapeutic approach for the treatment of silicosis of the lungs [26,27,28].
